# The Number of Liver Galectin-3 Positive Cells Is Dually Correlated with NAFLD Severity in Children

**DOI:** 10.3390/ijms20143460

**Published:** 2019-07-14

**Authors:** Felipe Leite de Oliveira, Nadia Panera, Cristiano De Stefanis, Antonella Mosca, Valentina D’Oria, Annalisa Crudele, Rita De Vito, Valerio Nobili, Anna Alisi

**Affiliations:** 1 Federal University of Rio de Janeiro, Institute of Biomedical Sciences, 21941-902 Rio de Janeiro, Brazil; 2 Molecular Genetics of Complex Phenotypes Research Unit, Bambino Gesù Hospital, IRCCS, 00146 Rome, Italy; 3 Histology-Core Facility, Bambino Gesù Hospital, IRCCS, 00146 Rome, Italy; 4 Hepato-Metabolic Disease Unit, Bambino Gesù Hospital, IRCCS, 00146 Rome, Italy; 5 Microscopy Unit, Bambino Gesù Hospital, IRCCS, 00146 Rome, Italy; 6 Histopathology Unit, Bambino Gesù Hospital, IRCCS, 00146 Rome, Italy

**Keywords:** non-alcoholic fatty liver disease (NAFLD), liver damage, Galectin-3 (Gal-3), inflammation, liver fibrosis, macrophage polarization

## Abstract

Non-alcoholic fatty liver disease (NAFLD) is a complex disease ranging from steatosis to non-alcoholic steatohepatitis (NASH). Galectin-3 (Gal-3), which is a β-galactoside binding protein, has been associated with liver fibrosis, but its role in NAFLD remains elusive. We investigated the expression of Gal-3 in liver resident cells and its potential association with liver damage in 40 children with biopsy-proven NAFLD. We found that several liver cells expressed Gal-3. The number of total Gal-3 positive cells decreased with the severity of disease and the cells were correlated with the presence of steatosis and the diagnosis of NASH. CD68 macrophages expressed Gal-3 but the number CD68/Gal-3 positive cells was significantly reduced in patients diagnosed with steatosis and NASH. Triple CD68/CD206/Gal-3, which represented the subpopulation of M2 macrophages, were mainly present in patients without NASH, and clearly reduced in patients with steatosis and NASH. On the contrary, the number of α-smooth muscle actin (SMA)/Gal-3 positive cells increased with the severity of fibrosis in children with NAFLD. Our data demonstrated that the number of Gal-3 positive cells was associated with tissue damage in different ways, which suggests a dual role of this protein in the pathogenesis of pediatric NAFLD, even if the role of Gal-3 deserves further studies.

## 1. Introduction

Non-alcoholic fatty liver disease (NAFLD), which is currently recognized as the most frequent cause of chronic liver disease worldwide, is a fatty liver condition occurring in the absence of alcohol consumption mainly associated with central obesity [1].

NAFLD comprises a large spectrum of histopathologic feature, which ranges from variable degrees of abnormal fat accumulation into hepatocytes (steatosis) to hepatic inflammation (non-alcoholic steatohepatitis, NASH) and eventually fibrosis [2]. More precisely, NASH is characterized by the coexistence of inflammation, hepatic cell injury (ballooning), and deposition of collagen fibers, which results in a specific histological pattern of hepatic disease. NASH carries the potential progression toward advanced liver disease, fibrosis, cirrhosis, and even hepatocellular carcinoma with consequence that, so far, NAFLD has become the second most common cause for liver transplantation in the USA adult population [3].

A recent meta-analysis estimated that the worldwide prevalence of NAFLD is around 25.24% in the adult population [4]. However, the raising of NAFLD is especially worrying in the pediatric population. Indeed, the results of a meta-analysis preformed in children and adolescents reported a 7.6% mean prevalence in the general population and a 34.2% prevalence in obese subjects [5].

In children, as well in adults, according to the “multiple hit model,” the factors that drive the onset and progression of NAFLD are likely heterogeneous, including metabolic factors (obesity, insulin resistance, and dyslipidemia), genetic and epigenetic factors, and lifestyle and environmental variables, and changes in crosstalk between different organs and tissues, like adipose tissue, pancreas, gut, and liver [6,7,8]. Overall, the full comprehension of the molecular mechanisms and factors that drive the hepatic inflammation in NAFLD remains a crucial issue to better define the pathogenesis and progression of liver damage and fibrosis during NAFLD [9].

Galectin-3 (Gal-3), which is a member of lectins protein able to bind galactose-containing glycoproteins on the cell surface and in the extracellular matrix, has been found to be implicated in various types of disease such as fibrosis, inflammation, and cancer [10,11,12]. In the liver, galectin-3 is absent in normal hepatocytes, but abundantly expressed in proliferating hepatocytes in cirrhotic liver [13].

In experimental chronic schistosomiasis, Gal-3 is expressed in inflammatory cells surrounding *Schistosoma mansoni*-eggs in the liver whereas Gal-3 knockout mice (Lgals3^−/−^ mice) showed significant disturbances in the hepatic collagen distribution and leukocyte mobilization [14]. Furthermore, Gal-3 expression was up-regulated in human fibrotic liver disease while its ablation inhibited myofibroblast activation attenuating liver fibrosis [15]. In particular, hepatic extracellular Gal-3 controls phagocytosis and activation in hepatic stellate cells (HSCs) [16].

The role of Gal-3 in the pathogenesis of NAFLD has been investigated by some experimental studies. Nomoto et al. (2006) reported that Lgals3^−/−^ mice developed clinico-pathological features similar to human NAFLD [17]. Experimental diet-induced NAFLD was more severe in the absence of Gal-3 with significant liver damages. These mice that were submitted to a high fat diet developed significant visceral adiposity, diabetes, and liver steatosis correlated with a reduced number of myeloid dendritic cells and proinflammatory macrophages [18,19]. During choline-deficient L-amino-acid-defined (CDAA) diet-induced NAFLD, Lgals3^−/−^ mice showed drastic hepatocellular injuries and pattern of gene expression associated with carcinogenesis and lipid metabolism [20].

In this study, we investigated whether Gal-3 expression in liver cells may be associated with tissue damage in children with biopsy-proven NAFLD.

## 2. Results

### 2.1. The Number of Gal-3 Positive Cells Correlates with the Severity of Disease in Children with NAFLD

Twenty-four male (60%) and 16 female (40%) patients with biopsy-proven NAFLD were included in this study. The main anthropometrical and metabolic characteristics of children are reported in Table 1.

Histological analysis revealed 22 (55%) children with NASH and 18 (45%) without NASH. Liver histological features of all children are shown in Table 2.

**Figure 1:**
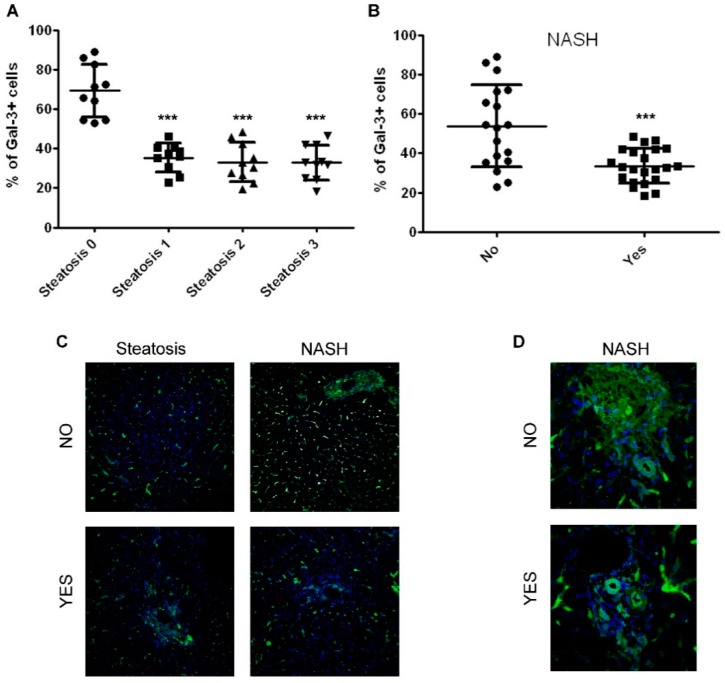
Analysis of the number of hepatic Gal-3 positive cells in children with NAFLD associated with the severity of disease. (**A**,**B**) Percentage of Gal-3 positive (Gal-3+) cells in the liver tissue from children with NAFLD correlated with degrees of steatosis (**A**) and the presence of NASH (**B**). *** *p* < 0.001 vs. steatosis 0 or no NASH. (**C**) Representative image of immunofluorescence of Gal-3 expression (green) in liver tissues from children with NAFLD showing the presence (Yes), or not (No), of steatosis and NASH. Magnification 20×. (**D**) Representative image of immunofluorescence of Gal-3 expression (green) in liver tissues from children with NAFLD, which shows the presence or absence of NASH in portal and lobular compartments. Magnification 60×. Nuclei were counteracted with Hoechst staining (blue).

Immunofluorescent analysis was performed in order to evaluate the number of Gal-3 positive (Gal-3+) cells in liver tissue of children with NAFLD. The percentage of Gal-3+ cells was significantly reduced in both patients with steatosis and NASH (Figure 1A–C). No difference was found in the number of Gal-3+ cells in patients with a distinct score for ballooning, fibrosis, and portal and lobular inflammation (Figure S1). Yet, morphological analysis (Figure 1D) showed that, in patients with NASH, both portal and lobular compartments were occupied by cells expressing Gal-3.

Moreover, Spearman correlations showed that the number of liver-resident cells that expressed Gal-3 were inversely correlated with alanine aminotransferase (ALT) (*r* = −0.31; *p* = 0.049) and aspartate aminotransferase (AST) (*r* = −0.31; *p* = 0.045) levels, NAFLD activity score (*r* = −0.57; *p* < 0.001), the presence of steatosis (*r* = −0.75; *p* < 0.001), and the diagnosis of NASH (*r* = −0.50; *p* = 0.001).

### 2.2. The Number of CD68/Gal-3 Positive Cells Decreases with Severity of Disease in Children with NAFLD

**Figure 2:**
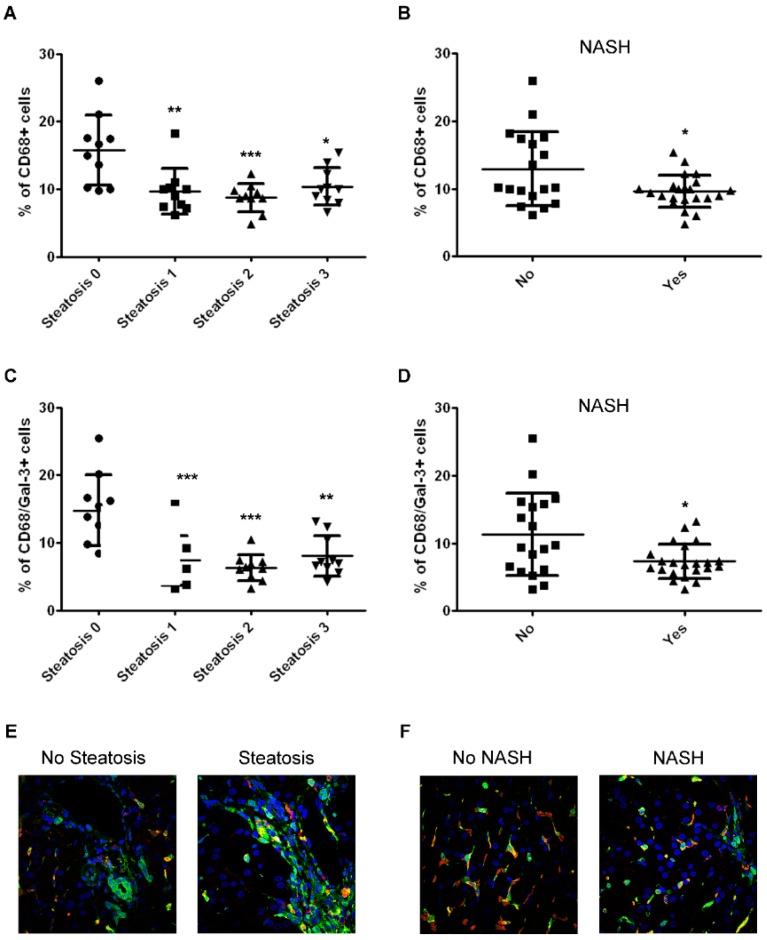
Analysis of the number of hepatic CD68/Gal-3 positive cells in children with NAFLD associated with the severity of disease. (**A**,**B**) Percentage of CD68+ cells in the liver tissue from children with NAFLD correlated with degrees of steatosis (**A**) and the presence of NASH (**B**). (**C**,**D**) Percentage of CD68/Gal-3+ cells in the liver tissue from children with NAFLD correlated with grades of steatosis (**C**) and the presence of NASH (**D**). *** *p* < 0.001; ** *p* < 0.01; * *p* < 0.05 vs steatosis 0 or no NASH. (**E**,**F**) Representative image of immunofluorescence of CD68 expression (red) and Gal-3 expression (green) in liver tissues from children with NAFLD showing the presence (steatosis), or not (No steatosis), of steatosis and NASH. Magnification 60×. Nuclei were counteracted with Hoechst staining (blue).

Since Gal-3 is highly expressed and secreted by macrophages [21], we evaluated the number of liver resident macrophages (CD68+) expressing Gal-3 in children with NAFLD and their correlation with severity of the disease. As shown in Figure 2A,B, the number of CD68+ macrophages was significantly reduced in patients with steatosis and NASH. No significant changes of the number of CD68+ cells were found in the different degrees of ballooning, fibrosis, and portal and lobular inflammation (Figure S2). Moreover, Spearman correlations showed that the number of liver-resident cells that expressed CD68 were inversely correlated with the presence of steatosis (*r* = −0.54, *p* < 0.001) and with NAS (*r* = −0.35, *p* = 0.05).

Accordingly to the trend of CD68 pattern, the number of CD68/Gal-3+ macrophages was also significantly reduced only in patients diagnosed with steatosis and NASH (Figure 2C,D). Morphological analysis revealed that CD68/Gal-3+ macrophages were preferentially observed in the hepatic portal zone of no steatosis group. In the course of steatosis, these double positive cells were found in a lower frequency (Figure 2E). On the other hand, the lobular region was hallmarked by CD68/Gal-3+ macrophages in no NASH group. The percentage of these cells was reduced in the liver of patients with NASH (Figure 2F).

### 2.3. The Number of CD68/CD206/Gal-3 Positive Cells Decreases with the Severity of Disease in Children with NAFLD

In order to investigate whether Gal-3+ macrophage reduction in patients with NASH was ascribable to a decrease of the number of Gal-3+ M2 macrophages, we performed a triple staining for CD68, Gal-3, and CD206 (human M2 marker).

**Figure 3:**
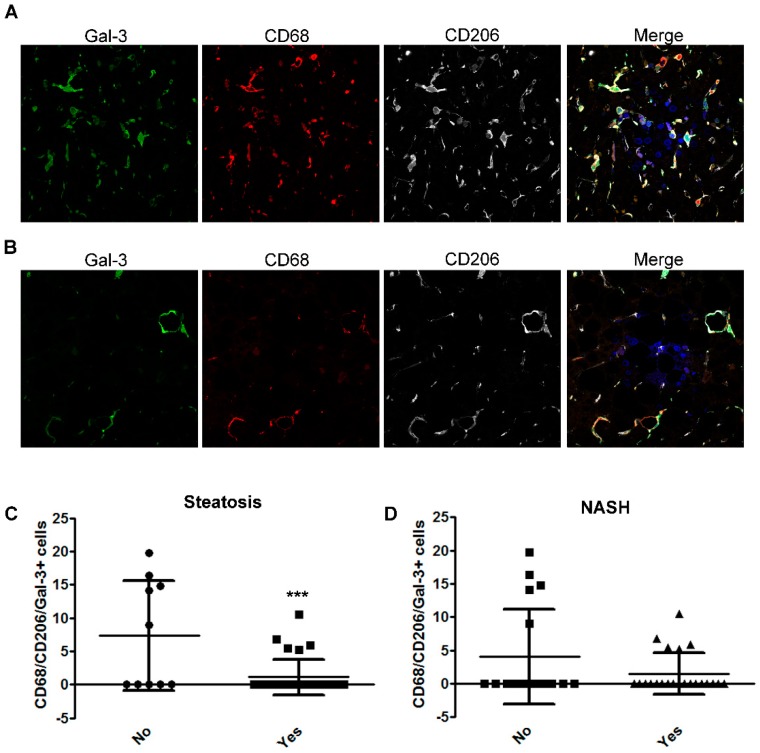
Analysis of the number of hepatic CD68/CD206/Gal-3+ cells in children with NAFLD associated with severity of disease. (**A**,**B**) Representative image of immunofluorescence of Gal-3 (green), CD68 (red) CD206 (grey) in liver tissues from children without NASH (**A**) and with NASH (**B**). Magnification 60×. Nuclei were counteracted with Hoechst staining (blue). (**C**,**D**) Mean number of CD68/CD206/Gal-3+ cells in the liver tissue from children with NAFLD correlated with the presence (Yes), or not (No) of steatosis and (**C**) NASH (**D**). *** *p* < 0.001.

In patients without NASH, most macrophages (approximately 80%) were triple positive (CD68/CD206/Gal-3+) and mainly located in the lobular region (Figure 3A). On the other hands, in NASH patients, the number of CD68/CD206/Gal-3+ macrophages were clearly reduced (Figure 3B). Quantitative analysis also revealed that the number of CD68/CD206/Gal-3+ macrophages was significantly reduced in patients with steatosis and exhibited a trend of decrease in NASH (Figure 3C,D).

Moreover, the number of CD68/CD206/Gal-3+ macrophages was significantly decreased in patients with grade 2 portal inflammation and with ballooning compared to children without these histologic features (Figure S3).

### 2.4. Bile Duct Cells Express Gal-3 in Children with NAFLD

The expression of Gal-3 was also investigated in hepatocytes by monitoring the double positivity of cells for Gal-3 and cytokeratin (CK)8/18 (marker of hepatocytes).

**Figure 4:**
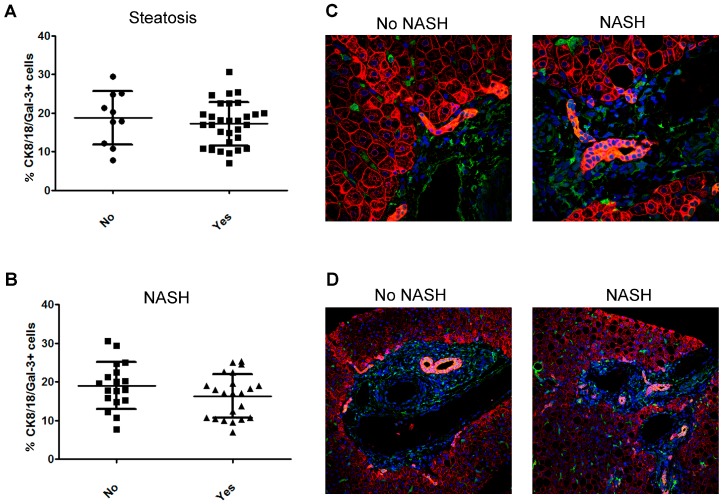
Analysis of the number of hepatic CK8/18/Gal-3+ cells in children with NAFLD associated with severity of disease. (**A**,**B**) Percentage of CK8/18/Gal-3+ cells in the liver tissue from children with NAFLD correlated with degrees of steatosis (**A**) and the presence of NASH (**B**). (**C**,**D**) Representative image of immunofluorescence of CK8/18 expression (red) and Gal-3 expression (green) in liver tissues from children with NAFLD in the presence (NASH) or not (No NASH) of NASH in the periportal zone (**C**) and bile duct (**D**). Magnification 60×. Nuclei were counteracted with Hoechst staining (blue).

No statically significant differences in the number of CK8/18/Gal-3+ hepatocytes were found in patients with steatosis vs. no steatosis, with NASH vs. no NASH (Figure 4A,B). The analysis of specific tissue area showed that, in the periportal zone, no NASH patients had a number of double-positive CK8/18/Gal-3+ hepatocytes higher than NASH children (Figure 4C). Moreover, a marked double co-localization of Gal-3 and CK8/18 staining in bile duct cells was also observed in both no NASH and NASH patients (Figure 4D).

### 2.5. The Number of α-Smooth Muscle Actin (SMA)/Gal-3+ Cells Increases with the Severity of Fibrosis in Children with NAFLD

Since the imaging data showed that HSCs expressed Gal-3, the assessment of the number of α-SMA/Gal-3+ cells was also performed.

**Figure 5:**
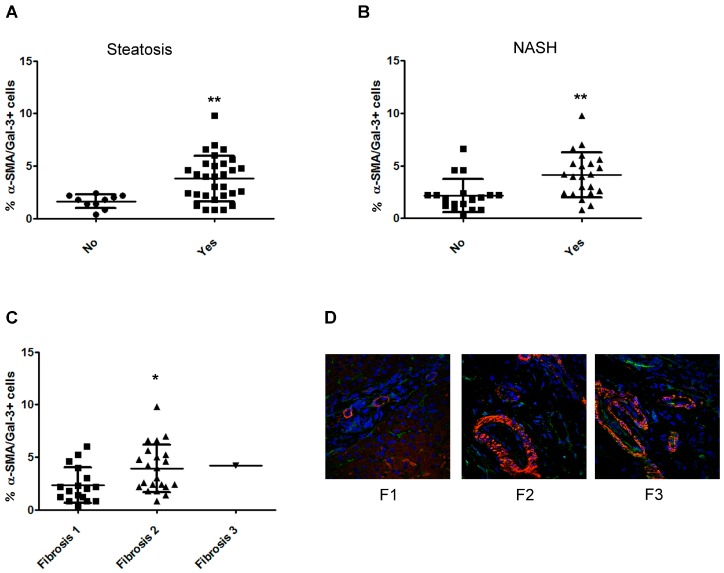
Analysis of the number of hepatic α-SMA/Gal-3+ cells in children with NAFLD associated with severity of fibrosis. (**A**–**C**) Percentage of α-SMA/Gal-3+ cells in the liver tissue from children with NAFLD correlated with degrees of steatosis (**A**), NASH (**B**), and the severity of fibrosis (**C**). (**D**) Representative image of immunofluorescence of α-SMA expression (red) and Gal-3 expression (green) in liver tissues from children with NAFLD with fibrosis scores of F1, F2, and F3. Magnification 60×. Nuclei were counteracted with Hoechst staining (blue). ** *p* < 0.01; * *p* < 0.05.

As shown in Figure 5A,B, patients with steatosis and NASH showed higher numbers of α-SMA/Gal-3+ cells than patients without these histologic features. Moreover, the number of α-SMA/Gal-3+ cells increased alongside the fibrotic score (Figure 5C,D).

## 3. Discussion

In this study, we demonstrate that liver resident cells express Gal-3. The number of hepatic Gal3+ cells decreases in children with liver steatosis and NASH. A large number of these Gal-3+ liver cells are M2 anti-inflammatory macrophages, and negatively correlated with the severity of liver damage. Yet, only a little portion of these Gal-3+ cells includes bile duct cells and HSCs. The number of the latter increases with the severity of fibrosis.

Gal-3, which is a β-galactoside binding protein, has been associated with fibrosis in distinct tissues, including the liver, while its role in the development of NAFLD is still controversial. Distinct experimental models have suggested that the lack of Gal-3 led to spontaneous NAFLD development. Lgals3^−/−^ mice aged six months showed significant liver disorders associated with high circulating levels of ALT, triglycerides, and liver lipid peroxide. Moreover, the same mice exhibited increased hepatic levels of advanced glycation end-products (AGE), receptor for AGE (RAGE), and peroxisome proliferator-activated receptor gamma (PPARγ) [17]. On the other hand, Iacobini et al. [22] reported that diet-induced NASH in Lgals3^−/−^ mice induced less severe liver damage in terms of steatosis, inflammation, and fibrosis. These discordant findings open questions on the real role of Gal-3 in NAFLD.

Gal-3 has also been indicated as a pro-inflammatory mediator, which is largely involved with macrophage activation and migration [11]. In the analgesic acetaminophen administration experimental model, Ly6C^low^ macrophages contained widely vacuolated cytoplasm, which is followed by an irregular shape and reduced mRNA levels of the pro-inflammatory proteins in comparison with Ly6C^high^ macrophages [23]. The same authors suggested that Gal-3 promoted persistent activation of F4/80+ pro-inflammatory Kupffer cells in centrilobular regions. Consistently, Lgals3^−/−^ mice showed reduced hepatotoxicity and inflammatory mediator production in response to acetaminophen [24].

Our data demonstrated that different liver cell subpopulations were Gal-3+, even if this protein was mainly expressed by the macrophage population. In fact, in the liver, the pan-macrophage marker CD68 was frequently co-expressed with Gal-3 and the number of double-positive cells was reduced in groups with steatosis, hepatocyte ballooning, and NASH. Thus, this suggests that the number of these cells was correlated with the severity of disease in children with NAFLD, and was confirmed by the inverse correlation with NAS.

It is well known that the hepatic M2 macrophage subpopulation is the most affected macrophage type in NAFLD because they play a protective effect against liver injury by promoting M1 macrophage apoptosis [9,25]. We previously demonstrated that the number of CD206+ cells, which represents M2 macrophages, was lower in NASH compared to not-NASH children [26]. Accordingly, we found that the CD68/CD206/Gal-3+ M2 macrophage number was reduced in patients with steatosis and NASH with a high grade of portal inflammation, or ballooning. These findings support a possible protective role of Gal-3+ M2 macrophages that we found impaired in children with NASH traits. In this case, a possible correlation with the NAFLD pathogenesis should be investigated at greater detail, considering that the combination between Kupffer cells, resident macrophages, and recruited bone marrow-derived macrophages can be critical for the establishment of NAFLD [27].

Our results revealed that the percentage of CK8/18/Gal-3+ hepatocytes in the lobular region and CK8/18/Gal-3+ bile duct cells in the portal region were unaffected by the severity of disease. However, we observed a reduction of the number of CK8/18/Gal-3+ cells in the periportal zones in NAFLD patients with steatosis and NASH. This type of cells was frequently observed during the ductular reaction and plays a critical role for the regeneration of the liver parenchyma [28]. Thus, it is plausible to suppose that Gal-3 is involved with liver parenchyma regeneration.

As widely reported in the literature, we found that the percentage of total α-SMA+ cells was significantly increased following the fibrosis stage [29,30]. Moreover, recently, Gal-3 has been described as an important biomarker to fibrosis in distinct organs [11]. Specifically, Gal-3 has been found to be expressed when activated by HSCs [15] and is mainly located in fibrotic zones in the liver [14,22,31]. Accordingly, we found that the number of α-SMA/Gal-3+ cells was significantly increased following the fibrosis stage. Our data reinforced the literature in this question, considering that a direct correlation between an increased fibrosis score and α-SMA/Gal-3+ cells in NAFLD children could reveal a pro-fibrogenic role of Gal-3.

Our study reinforces the evidence that Gal-3 might be evaluated as a potential therapeutic target for NAFLD in the preclinical and clinical study that point to a reduction in fibrosis. The preclinical study reported that the administration of GR-MD-02, which is a galectin-3 inhibitor, was able to reduce collagen fiber deposition in both the mouse NASH model and cirrhotic rats [32,33]. Recently, proofs of tolerability and pharmacodynamic effects of GR-MD-02 have supported the start of a phase-2 randomized clinical trial that is currently ongoing [34]. However, it remains that defining this type of treatment could be detrimental for the tissue inflammatory state.

In conclusion, our findings demonstrated that the number of Gal-3 positive cells associated with tissue damage in different ways suggests a dual role of this protein in the pathogenesis of pediatric NAFLD, even if the role of Gal-3 deserves further studies that could help plan future studies of pharmacological targeting of this molecule.

## 4. Materials and Methods

### 4.1. Patients

This study includes 40 children with biopsy-proven NAFLD properly registered and admitted at the Hepato-Metabolic Disease Unit of the Bambino Gesù Children’s Hospital and IRCCS (Rome, Italy) from September 2016 to January 2017. All protocols followed the ethical guidelines of the 1975 Declaration of Helsinki and this study was approved by Bambino Gesu` Children’s Hospital local ethics committee (protocol number: 539/RA, 01/07/2013). Written informed consent was obtained from the parents of each child.

All included children were Caucasians of Italian descent. None of them were taking any medication. All children were tested for secondary causes of hepatic steatosis, such as alcohol abuse, total parenteral nutrition, and chronic use of drugs known to induce hepatic steatosis (e.g., valproate, amiodarone, and prednisone). Hepatitis A, B, and C, cytomegalovirus, Epstein-Barr virus infections, and celiac disease were excluded, according to appropriate serological tests. Autoimmune liver disease, metabolic liver disease, Wilson’s disease, and alpha-1-antitrypsin-associated liver disease were ruled out using standard clinical, laboratory, and histological criteria.

### 4.2. Anthropometrics

Body mass index (BMI) was measured as kilograms divided by the square of height in meters and then the standardized BMI z-score was also calculated. Waist circumference was measured with the patient in a standing position on the horizontal plan between the lowest portion of the rib cage and the iliac crest [35,36].

### 4.3. Laboratory Tests

Venous blood samples were collected in the morning after an overnight fast of at least 8 h and immediately processed. Serum glucose, AST, ALT, gamma-glutamyltransferase (GGT), total triglycerides and cholesterol, low-density lipoprotein (LDL), high-density lipoprotein (HDL), and insulin were measured using standard laboratory procedures. The homeostasis model assessment (HOMA-IR) score was used for estimating insulin resistance, according to the equation [fasting insulin (μU/mL) X fasting glucose (mg/dL)/405] [37].

### 4.4. Liver Biopsies

All children underwent a liver biopsy for a persistently elevated serum aminotransferase level and/or diffusely hyperechogenic liver on ultrasonography and after exclusion of other common or less common liver diseases described above.

Echo-guided liver biopsies were fixed in 10% buffered formalin and steatosis, lobular inflammation, hepatocyte ballooning, and fibrosis were scored, according to criteria proposed by the NAFLD Clinical Research Network [38]. Steatosis was graded 0–3 (0 ≤ 5% steatosis, 1 = 5–33%, 2 = 33–66%, and grade 3 ≥ 66%). Lobular inflammation was scored based on the number of inflammatory foci per 200× per field (0  =  no inflammatory foci, 1  ≤ 2 foci, 2  =  2–4 foci and 3  ≥ 4 foci), ballooning was graded 0–2 (0  =  none, 1  =  few balloon cells present, and 2  =  prominent ballooning). An NAFLD activity score (NAS) > 5 was used for further comparisons with variables of interest.

Fibrosis was staged 0–4 as follows: 0 = no fibrosis; 1 = periportal or perisinusoidal; 1A = mild, Zone 3, perisinusoidal; 1B = moderate, Zone 3, perisinusoidal; 1C = portal/periportal; 2 = perisinusoidal and portal/periportal; 3 = bridging fibrosis, and 4 = cirrhosis.

Portal inflammation (0 = no PI, 1 = mild PI, 2 = more than mild) as previously defined and specifically for children [39].

Diagnosis of NASH or NAFLD instead of NASH was made by an experienced pathologist based on the aggregate presence and degree of the individual features of NAFLD.

### 4.5. Immunofluorescence (IF) Staining

A double or triple IF staining were performed on 2 μm-thick sections obtained from formalin-fixed tissue embedded in paraffin of biopsy-proven NAFLD patients. Antigen retrieval was performed with ethylenediaminetetraacetic acid (EDTA) (pH 9) (Dako, Glostrup, Denmark).

Liver samples were stained after overnight incubation at +4 °C with anti-human: goat polyclonal anti-Galectin-3 antibody (diluition 1:200 R&D Systems, Minneapolis, MN, USA), mouse monoclonal anti-α-SMA antibody (dilution 1:100 Dako, Glostrup, Denmark), mouse monoclonal anti-Cytokeratin-8/18 (diluition 1:100 Monosan, Uden, The Netherlands), mouse monoclonal anti-CD68 (diluition 1:200 Abcam, Cambridge, UK), and rabbit monoclonal anti-CD206 (diluition 1:500 Abcam, Cambridge, UK). After incubation with primary antibodies, specimens were washed and treated for 1 h with labeled isotype-specific secondary antibodies, mouse anti-goat Alexa Fluor 488, goat- anti-mouse Alexa Fluor 555 (1:500, Applied Biosystems, Life Technologies, Carlsbad, CA, USA), and mouse anti-rabbit Alexa Fluor 488 (1:500, Life Technologies, Carlsbad, CA, USA). Nuclei were counterstained with Hoechst. The sections were mounted with PBS/glycerol (1:1) and covered with a coverslip.

Images were acquired using the Olympus Fluoview FV1000 confocal microscope, equipped with a 20× (N.A. 0.75) and 60× (N.A. 1.42 oil objective) objectives. Optical single sections were acquired with a scanning mode format of 1024 × 1024 pixels, sampling speed of 20 μs/pixel, and 12 bits/pixel images. Fluorochromes unmixing was performed by acquiring automated-sequential collection of multi-channel images, in order to reduce spectral crosstalk between channels.

Before manual counting, images were cropped, scaled to μm, and separated by color channel, and artifacts were removed. Next, two independent technicians (CDS and VDO) performed the manual count of the images.

### 4.6. Statistics

Descriptive statistics were calculated for variables of interest by using Graphpad Prism 5.01 software. The student t test and Anova were properly used to determine statistical significance between groups. A Spearman correlation was performed to measure the strength of the linear relationships. Statistical significance was set at *p* ≤ 0.05.

## Figures and Tables

**Table 1:** Anthropometrical and metabolic patient’s characteristics (*n* = 40).

Parameters	Mean	S.D.
Age (years)	12.36	2.88
Weight (kg)	66.60	17.77
Height (cm)	153.1	16.42
BMI (kg/m^2^)	28.00	5.37
Waist Circumference (cm)	85.83	8.51
Triglycerides (mg/dl)	119.3	63.04
Cholesterol (mg/dl)	154.8	33.89
HDL-cholesterol (mg/dl)	45.04	8.77
LDL-cholesterol (mg/dl)	95.46	32.65
ALT (IU/L)	47.93	39.80
AST (IU/L)	33.85	16.74
GGT (IU/L)	20.50	15.11
Glucose (mg/dl)	82.60	10.39
Insulin (U/L)	21.20	10.25
HOMA-IR	4.19	2.21

**Table 2:** Histological features of patients (*n* = 40).

Histologic Traits	Mean of Scores	S.D.
Steatosis	1.50	1.13
Lobular Inflammation	1.15	0.42
Portal Inflammation	1.15	0.42
Ballooning	1.02	0.57
NAS	3.70	1.53

## Supplementary Materials

Supplementary materials can be found at https://www.mdpi.com/1422-0067/20/14/3460/s1.

## Abbreviations

ALT	Alanine aminotransferase
AST	Aspartate aminotransferase
BMI	Body mass index
Gal-3	Galectin-3
GGT	Gamma-glutamyltransferase
HDL	High-density lipoprotein
HOMA-IR	Homeostasis model assessment
HSCs	Hepatic stellate cells
LDL	Low-density lipoprotein
NAFLD	Non-alcoholic fatty liver disease
NAS	NAFLD activity score
NASH	Non-alcoholic steatohepatitis
