# Supplementary material for: The Number of Liver Galectin-3 Positive Cells Is Dually Correlated with NAFLD Severity in Children
Source: Int J Mol Sci. 2019 Jul 14;20(14):3460. doi: 10.3390/ijms20143460 (PMC6679049; doi:10.3390/ijms20143460)
Supplement: Supplementary file 1 [file ijms-20-03460-s001.pdf]

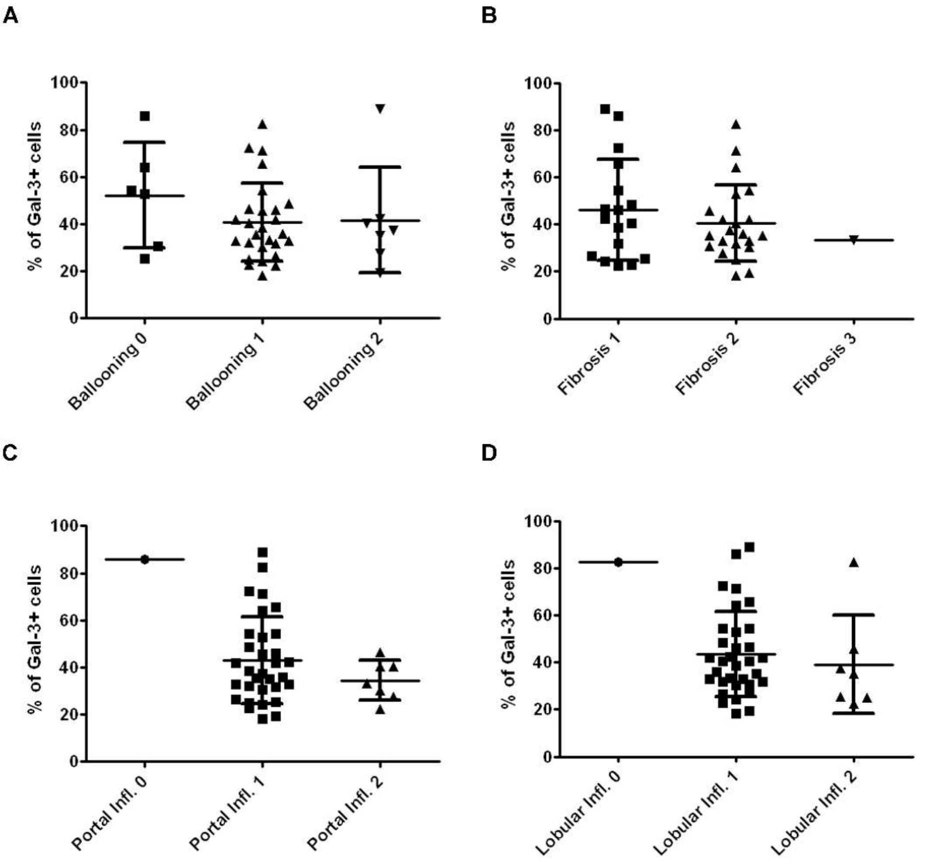

Figure S1. Percentage of hepatic Gal-3 + cells in children with NAFLD associated with the severity of disease. (**A–D**) Percentage of Gal-3 positive (Gal-3+) cells in the liver tissue from children with NAFLD correlated with degrees of ballooning (**A**), fibrosis (**B**), portal inflammation (**C**), and lobular inflammation (**D**).

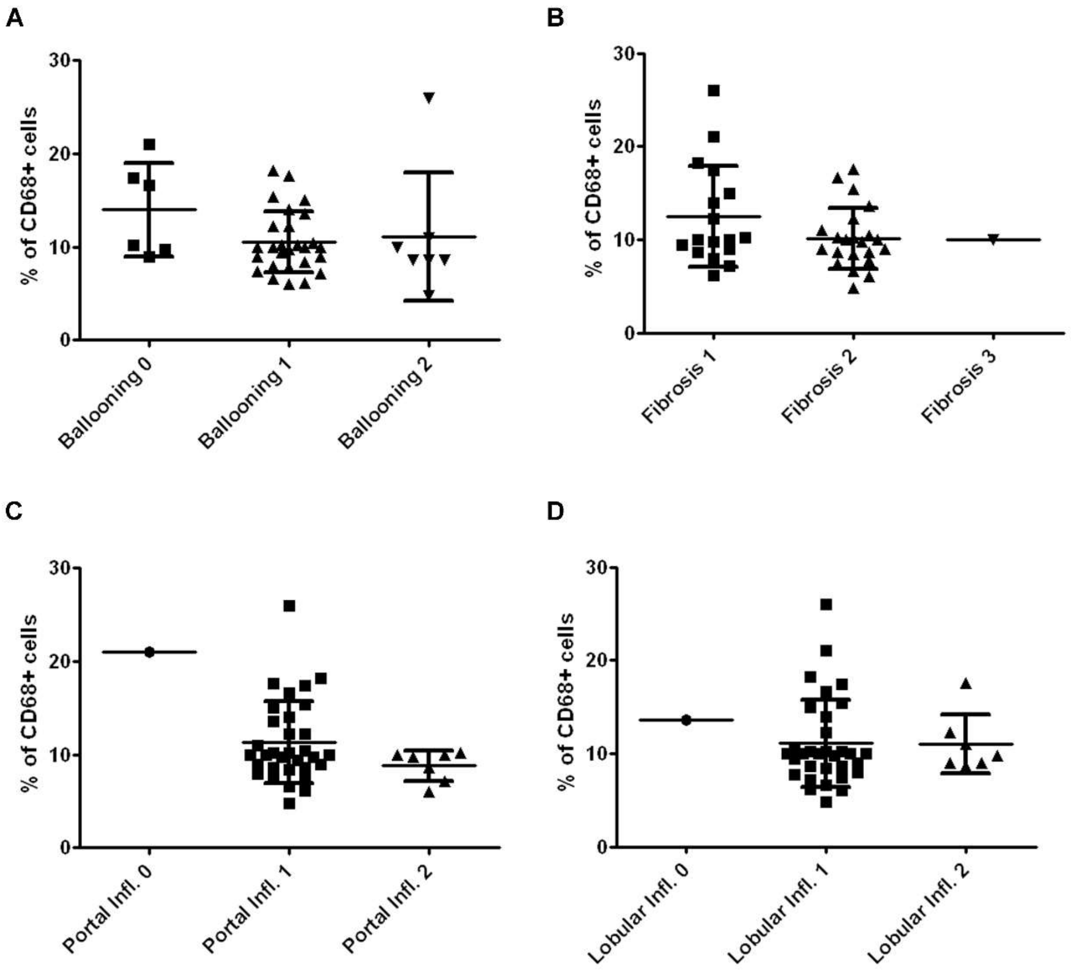

Figure S2. Percentage of hepatic CD68+ cells in children with NAFLD associated with the severity of disease. (**A**–**D**) Percentage of Gal-3 positive (Gal-3+) cells in the liver tissue from children with NAFLD correlated with degrees of ballooning (**A**), fibrosis (**B**), portal inflammation (**C**), and lobular inflammation (**D**).

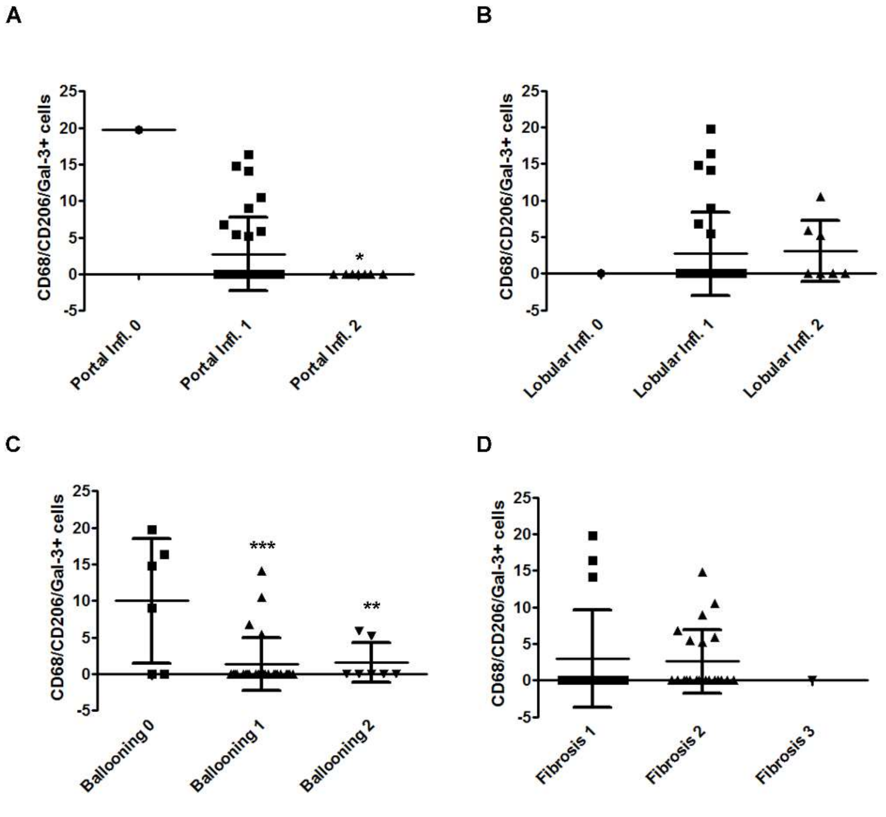

Figure S3. Mean number of hepatic CD68/CD206/Gal-3+ cells in children with NAFLD associated with the severity of disease. (**A**–**D**) Percentage of Gal-3 positive (Gal-3+) cells in the liver tissue from children with NAFLD correlated with degrees of portal inflammation (**A**), lobular inflammation (**B**), ballooning (**C**), and fibrosis (**D**).
